# Automated localization of the medial clavicular epiphyseal cartilages using an object detection network: a step towards deep learning-based forensic age assessment

**DOI:** 10.1007/s00414-023-02958-7

**Published:** 2023-02-02

**Authors:** Philipp Wesp, Bastian Oliver Sabel, Andreas Mittermeier, Anna Theresa Stüber, Katharina Jeblick, Patrick Schinke, Marc Mühlmann, Florian Fischer, Randolph Penning, Jens Ricke, Michael Ingrisch, Balthasar Maria Schachtner

**Affiliations:** 1 Department of Radiology, University Hospital, LMU Munich, Marchioninistraße 15, 81377 Munich, Germany; 2 Comprehensive Pneumology Center (CPC-M), Member of the German Center for Lung Research (DZL), Max-Lebsche-Platz 31, 81377 Munich, Germany; 3 Institute of Informatics, LMU Munich, Oettingenstraße 67, 80538 Munich, Germany; 4 Institute of Forensic Medicine, LMU Munich, Nußbaumstraße 26, 80336 Munich, Germany

**Keywords:** Anatomic landmark detection, Deep learning, Object detection, Medial clavicular epiphyseal cartilages, Age assessment

## Abstract

**Background:**

Deep learning is a promising technique to improve radiological age assessment. However, expensive manual annotation by experts poses a bottleneck for creating large datasets to appropriately train deep neural networks. We propose an object detection approach to automatically annotate the medial clavicular epiphyseal cartilages in computed tomography (CT) scans.

**Methods:**

The sternoclavicular joints were selected as structure-of-interest (SOI) in chest CT scans and served as an easy-to-identify proxy for the actual medial clavicular epiphyseal cartilages. CT slices containing the SOI were manually annotated with bounding boxes around the SOI. All slices in the training set were used to train the object detection network RetinaNet. Afterwards, the network was applied individually to all slices of the test scans for SOI detection. Bounding box and slice position of the detection with the highest classification score were used as the location estimate for the medial clavicular epiphyseal cartilages inside the CT scan.

**Results:**

From 100 CT scans of 82 patients, 29,656 slices were used for training and 30,846 slices from 110 CT scans of 110 different patients for testing the object detection network. The location estimate from the deep learning approach for the SOI was in a correct slice in 97/110 (88%), misplaced by one slice in 5/110 (5%), and missing in 8/110 (7%) test scans. No estimate was misplaced by more than one slice.

**Conclusions:**

We demonstrated a robust automated approach for annotating the medial clavicular epiphyseal cartilages. This enables training and testing of deep neural networks for age assessment.

## Background

Age is an essential part of a person’s identity, especially for children. By definition of the UN Convention on the Rights of the Child (CRC, Article 1) [1] and the EU acquis (Directive 2013/33/EU, Article 2(d)) [2], a child is any person below the age of 18. When the age is known, it rules the relationship between a person and the state. Changes in age can trigger the acquisition of rights and obligations in different aspects such as emancipation, employment, criminal responsibility, sexual relation, and consent for marriage or military service [3]. Because of the importance of age, the CRC lists certain key obligations for states and authorities regarding age that include registration of the child after birth, respecting the right of the child to preserve his or her identity, and speedily re-establish his or her identity in case that some or all elements of the child’s identity have been deprived [3]. Following these obligations, a state may need to assess the age of the person to determine whether the person is an adult or a child when the age is unknown. In that case, the European Union Agency for Asylum (EUAA) recommends that the least intrusive method is selected following a gradual implementation and that the most accurate method is selected and margin of error is documented [3].

Radiological examinations of the carpal bones, the molars or the clavicles play an important role in assessing the chronological age of living individuals [4]. For the clavicles, the ossification status of the medial clavicular epiphyseal cartilages is of particular interest. As the last maturing bone structure in the body, it allows age assessment not only for minors, but also for young adults [5]. However, current standard methods for age assessment suffer from low accuracy, intra- and inter-reader variability, and low diversity within the study populations [4, 6, 7].

A promising approach for accurate and automated age assessment is deep learning. Deep learning has been applied successfully to a wide range of computer vision tasks in medical imaging in the past [8–10]. A deep neural network trained to map an image of the medial clavicular epiphyseal cartilages to an individual’s age may yield more accurate age assessment results compared to current approaches [8, 11, 12]. The data required to train a deep network for age assessment, i.e., medical images including clavicles and sternum, as well as information about the age of the corresponding individuals, is abundant in many hospitals and also easily accessible. However, for efficient and successful training, it is advisable to first localize the medial clavicular epiphyseal cartilages within the medical images. The training process for diagnostic computer vision networks benefits from inputs that are cropped to the image region containing information relevant for solving the problem [13]. This cropping step usually requires manual expert annotations, which are time-consuming and expensive [14].

Therefore, the aim of this study was to develop and to evaluate an automated approach to localize the medial clavicular epiphyseal cartilages in CT scans, using deep learning–based object detection. This automated localization can be used to create large datasets, which are necessary to appropriately train and evaluate a deep neural network for age assessment [15].

## Methods

**Fig. 1:**
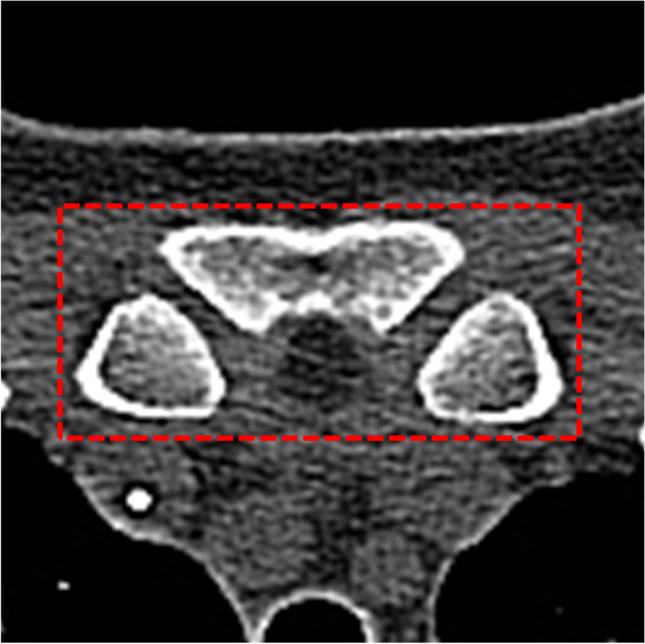
The structure-of-interest (SOI), defined as the sternoclavicular joints, together with their contributing portions of the sternum and the medial clavicles

**Fig. 2:**
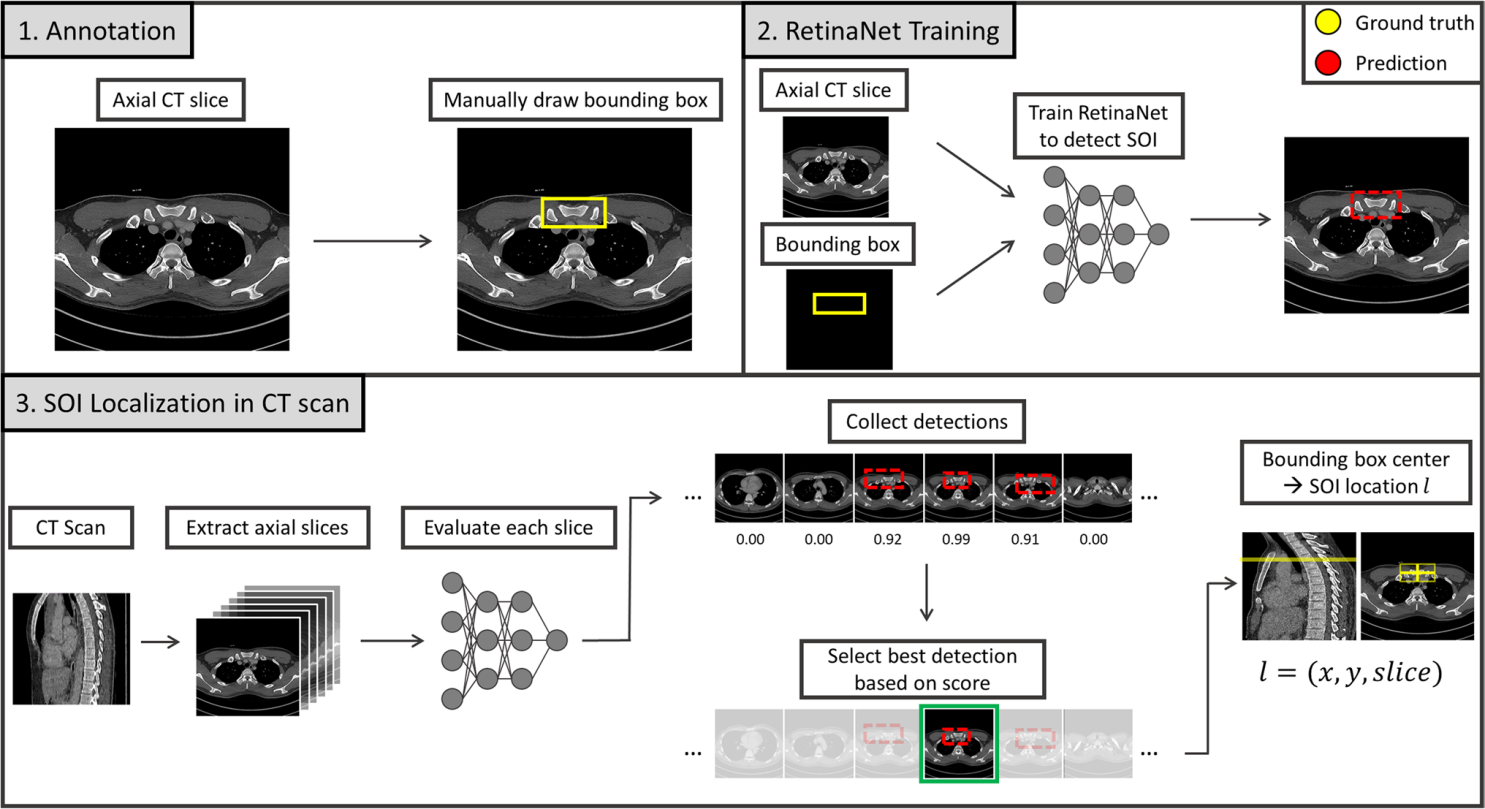
Schematic workflow diagram of the proposed medial clavicular epiphyseal cartilage localization. 1. Annotation: CT images are manually annotated with two-dimensional ground-truth bounding boxes in axial slices around the structure-of-interest (SOI). The SOI is an easy-to-identify proxy structure for the actual medial clavicular epiphyseal cartilage. 2. RetinaNet Training: A RetinaNet is trained to detect the SOI in axial slices and predict bounding boxes. 3: Localization in CT scan: The SOI can be localized in an unknown CT scan of the upper body. For this purpose, the trained RetinaNet is applied to each slice in a CT scan and all positive detections are collected. Afterwards, the center of the bounding box which corresponds to the best detection (highest classification score) is used as the predicted location for the SOI

We propose to use the state-of-the-art object detection network RetinaNet [16] for the automated localization of the medial clavicular epiphyseal cartilages in CT scans. First, a trained instance of the two-dimensional RetinaNet was applied to each axial slice in a scan in order to detect a proxy structure for the medial clavicular epiphyseal cartilages (Fig. 1). In case of a detection, the RetinaNet predicted a bounding box, as well as a class, and provided a classification score. Multiple detections in different slices or within the same slice were possible. The center of the bounding box associated with the highest of all classification scores was entitled as the location estimate of the medial clavicular epiphyseal cartilages in the CT scan. The entire workflow is illustrated in Fig. 2.

In the following, this section will describe (a) the retrospective collection of the data; (b) the manual data annotation; (c) the splitting of the data into training, validation, and test set; (d) the object detection network RetinaNet; (e) the training and evaluation of the RetinaNet; and (f) how we used the RetinaNet to estimate the location of the medial clavicular epiphyseal cartilages in a scan.

### Retrospective data collection

This study was approved by the institutional review board, and the requirement for written informed consent was waived. CT scans of the upper body were identified retrospectively in the picture archiving and communication system (PACS). The scans were originally acquired during the clinical routine for all purposes in the period 2017–2020. The patients’ age at examination was in the range of 15 to 25 years; age was measured as the time difference in days between documented date of birth and date of CT examination. On the one hand, this range covers a broad spectrum of developmental stages of the medial clavicular epiphyseal cartilages [17]; on the other hand, it includes ages which have high legal relevance in most countries, e.g., R18 and 21 [4]. Detailed inclusion and exclusion criteria for CT scans are listed in the “Appendix.”

Three preprocessing steps were applied to the collected scans. First, image voxel values were limited to the range of − 200 to 600 Hounsfield units (HUs). This value range was derived heuristically, with the intent to remove information from the image that we considered less relevant for the detection of the proxy structure for the medial clavicular epiphyseal cartilages. This signal intensity restriction was supposed to guide the network to focus on a balanced mix of bone and clavicles surrounding soft tissue. Second, axial slices have been resized to 512 × 512 pixels to match the input size that is expected by the RetinaNet. Finally, pixel values in each axial slice were linearly scaled into the value range 0.0 to 1.0 for network training.

### Manual data annotation

The structure-of-interest (SOI) in this study was defined as the sternoclavicular joints, together with their contributing portions of the sternum and the medial clavicles (Fig. 1). The SOI served as an easy-to-identify proxy for the actual medial clavicular epiphyseal cartilages and was to be the structure detected by the RetinaNet.

**Fig. 3:**
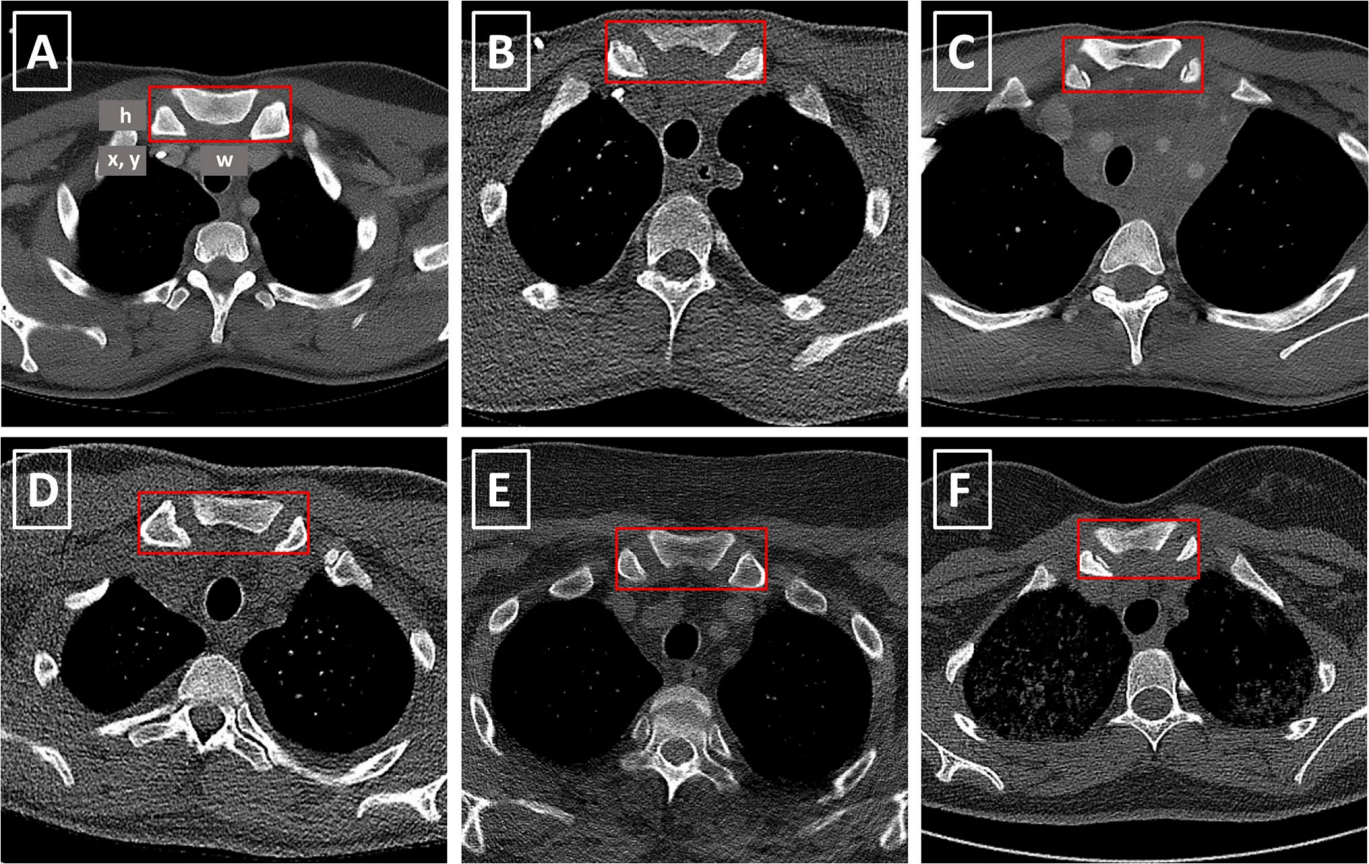
(**A**–**F**) Bounding boxes around the SOI in different CT scans after preprocessing. The SOI is defined as the sternoclavicular joints, together with their contributing portions of the sternum and the medial clavicles. In addition, (**A**) illustrates the 4 bounding box location parameters x, y, width (w), and height (h)

All axial slices from the collected CT scans were manually annotated with ground-truth target labels for the RetinaNet. These target labels have two components: first, a bounding box which located the object, represented by 4 parameters—(a) *x* position, (b) *y* position, (c) width, and (d) height—and second, a class label which classified the object. If a slice included the SOI, a target label was created by manually drawing a bounding box around all visible portions of the sternum and the medial clavicles contributing to the sternoclavicular joints and setting the class label of the object to “sternum” (Fig. 3). Depending on the patient and scanning protocol, in particular the slice thickness, multiple consecutive axial slices contained the SOI and were annotated with bounding boxes and class labels accordingly.

### Training, validation and test set

The network was evaluated using the three-way holdout method. To this end, the *n* = 222 collected and annotated CT scans were split into three sets: (a) training, (b) validation, and (c) testing. First, a test set consisting of 110 scans from 110 patients was randomly selected from the whole study dataset, so that it contained *n* = 10 patients and scans per age in years (ages = 15, 16, …, 25). All remaining *n* = 112 scans that have not been selected for the test set were split according to a 90/10 ratio into a training set of *n* = 100 (= Floor[0.9 × 112]) scans and a validation set of *n* = 12 (= 112—100) scans. The test set was used only for the evaluation of the RetinaNet, once the training was completed. The training set was used to train the RetinaNet, while the validation set was used to monitor the training process. No resampling strategy, such as cross-validation, was applied.

### Object detection network

The automated localization of the SOI proposed in this study was based on a PyTorch implementation [18, 19] of the object detection network RetinaNet [16]. This RetinaNet had a ResNet18 [20] backbone, which was provided by PyTorch as an off-the-shelf network and had been pre-trained on the public dataset ImageNet [21]. An important component of the RetinaNet implementation was the Focal Loss [16], which addressed heavy class imbalance for one-stage object detectors like the RetinaNet. This was useful, as the majority of an upper body CT scan does not cover the SOI.

As input, the network expects an image size of 512 × 512 pixels, being a two-dimensional axial slice from a preprocessed CT scan. In case of a detection, the network returns three outputs: (a) a bounding box prediction which locates the detected object, (b) a class prediction which classifies the detected object, and (c) a classification score between 0.0 and 1.0 quantifying the confidence of the network in the predicted detection. The class prediction is trivial, as “sternum” is the only class. Higher classification scores imply increased confidence in the detection.

### Object detection training and evaluation

The RetinaNet was trained for 20 epochs with examples from the training set using the Adam optimization algorithm [22] and a base learning rate of 10^–5^ to minimize the focal loss. A learning-rate scheduler decreased the learning rate by a factor of 10 whenever the loss did not improve for 3 consecutive epochs. Data augmentation was applied: during training, we randomly flipped the image input and the bounding box along the same randomly chosen axis. Training progress was monitored by evaluating the loss of the validation set.

After training, the RetinaNet was applied to and evaluated on the test set. The predicted bounding boxes and class labels were compared to the manually annotated ground-truth targets to identify positive and negative detections—the term negative detection can be used interchangeably with no detection. A classification score ≥ 0.05 is considered a positive detection. The detection is true positive, if the intersection over union (IoU) for the areas of the predicted bounding box ${A}_{pred}$ and the ground-truth bounding box ${A}_{true}$ is > 0.5 and the predicted class is the ground-truth class. Otherwise, the detection is considered false positive.

$$IoU\left({A}_{pred}, {A}_{true}\right)=\frac{\left|{A}_{pred}\cap {A}_{true}\right|}{\left|{A}_{true}\cup {A}_{pred}\right|}$$

A classification score < 0.05 (value adapted from [16]) is a true-negative detection, if the image does not contain a bounding box labeled as “sternum.” Otherwise, it is a false-negative detection.

Network performance was evaluated using average precision (AP) [23], a popular metric for object detection since it was applied for the PASCAL Visual Object Classes (VOC) Challenge in 2007 [24, 25]. AP is calculated as the area under the precision-recall curve from all positive and negative network detections for the test set, ranked according to classification score in descending order, where the precision $p$ is set to the maximum precision obtained for any recall ${r}^{\mathrm{^{\prime}}}\ge r$ [25]:

$$AP = \sum_{n}({r}_{n+1}-{r}_{n})\cdot {p}_{interp}({r}_{n+1})$$

$${p}_{interp}\left({r}_{n}\right)=\max{p\left({r}^{\mathrm{^{\prime}}}\right)},\quad{r}^{\mathrm{^{\prime}}}:r\mathrm{^{\prime}}\ge {r}_{n}$$

### Estimating the location of the SOI

The RetinaNet was trained to detect the presence of the SOI in axial CT slices. Because the SOI is a structure which typically stretches across multiple axial slices in a CT, the network may return positive detections for multiple slices. However, for data annotation purposes, we wanted the localization approach to yield a unique location estimate of the SOI for a given CT scan.

Estimating the location of the SOI included the following steps (see Fig. 2): (a) apply the RetinaNet to each slice in a given CT scan, (b) collect all positive detections, (c) select the best detection based on the classification score, and (d) select the center of the bounding box of the best detection to be the unique estimated location of the SOI. For example, when given a CT scan consisting of 300 axial slices, the RetinaNet may detect the SOI in slices 240 and 241. The detection in slice 241 may have a classification score of 0.96, while slice 240 may only have a score of 0.92. In that case, the bounding box center of the detection in slice 241 would be the unique estimated location of the SOI. In this context, a location encoded the position in three dimensions (x, y, slice): the position in the axial plane (x, y) and the number of the slice of the respective detection counting in axial direction (slice).

Location estimates were evaluated per scan. Location estimates based on true-positive detections were also true positives. Location estimates based on false-positive detections were also false positives. Location estimates for scans with no positive detection were automatically false negatives, because each scan contained the SOI. There were no true-negative location estimates. We also evaluated the Euclidean distance between the estimated location and the center of the (nearest) ground-truth bounding box in the axial plane. Additionally, we evaluated the number of slices between the estimated location and the center of the (nearest) ground-truth bounding box.

## Results

### Data

The retrospectively collected image data (training set, validation set, and test set) in this study comprised 63,999 two-dimensional axial slices from 222 CT scans and 202 patients (86 female (42.6%)) from age 15 to 25. In total, 872/63,999 (1.4%) slices include the SOI and were annotated with the class label “sternum” and with a ground-truth bounding box.

The total image data was divided into three sets: training set, validation set, and test set (Table 1). The test set consisted of 30,846 slices from 110 scans and 110 patients (50 female (45.5%)); 379/30,846 (1.2%) slices included the SOI, were labeled as class “sternum,” and had a ground-truth bounding box. The training set consisted of 29,656 slices from 100 scans and 82 patients (35 female (42.7%)); 434/29,656 (1.5%) slices included the SOI, were labeled as class “sternum,” and had a ground-truth bounding box. The validation set consisted of 3497 slices from 12 scans and 10 patients (1 female (10.0%)); 41/3,497 (1.2%) slices included the SOI, were labeled as class “sternum,” and had a ground-truth bounding box.

**Table 1:** Training, validation, and test set composition with respect to the number of patients, CT scans, axial slices, and annotated slices

Dataset	Patients	Scans	Slices	Annotated slices
Training	82	100	29,656	434 (1.5%)
Validation	10	12	3,497	41 (1.2%)
Test	110	110	30,846	379 (1.2%)

### Object detection network

The trained RetinaNet achieved an AP of 0.82 (1.0 = perfect score) for the detection, i.e., simultaneous localization and classification, of the SOI in the two-dimensional axial CT scan slices of the test set. The average IoU of the bounding boxes predicted by the RetinaNet and the ground-truth bounding boxes was 0.74 (1.0 = identical boxes; 0.0 = no overlap between boxes).

**Fig. 4:**
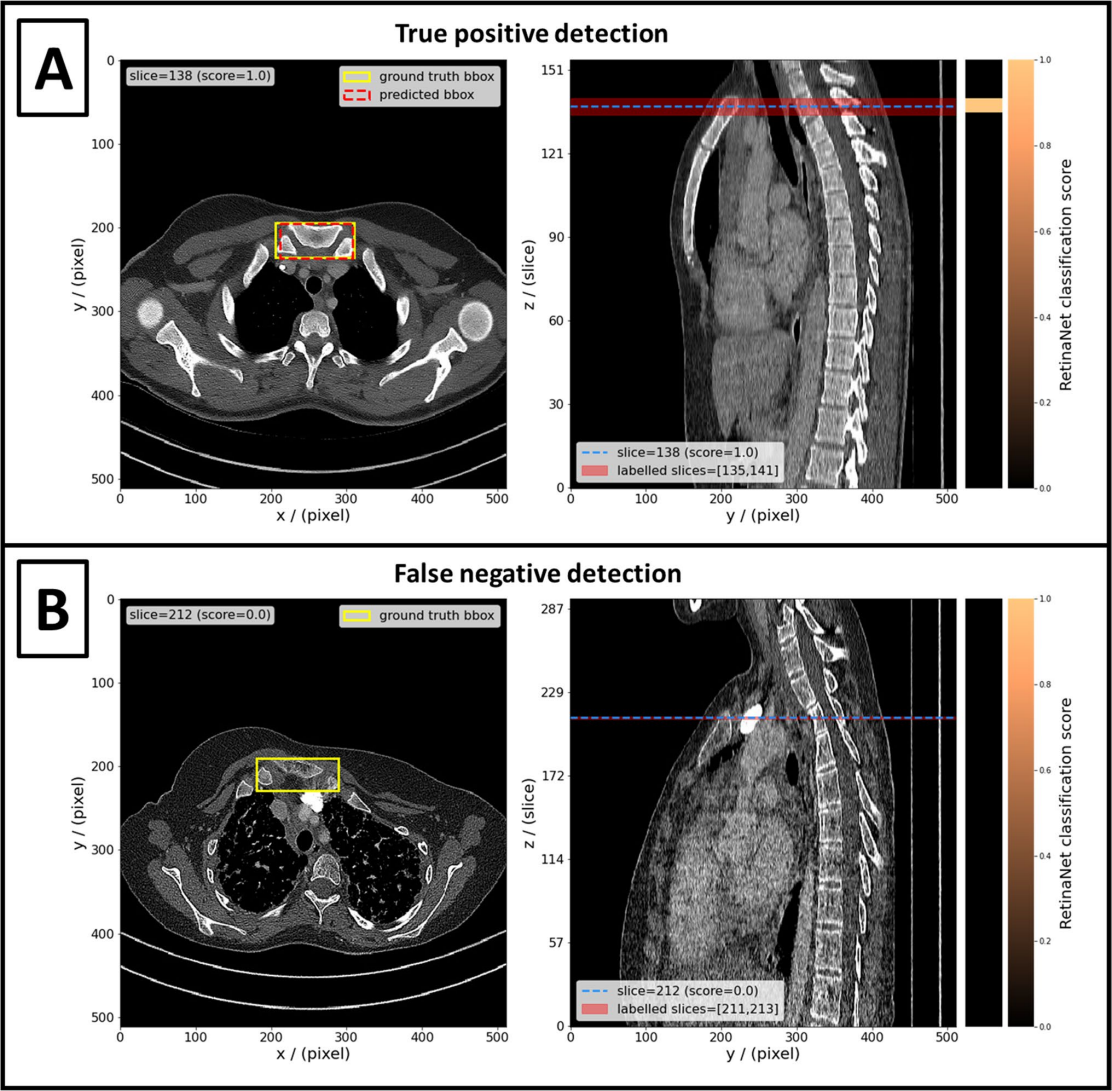
The left panels show axial CT slices with ground-truth bounding boxes around the SOI (yellow boxes) and detections (if predicted by network) (red boxes). The right panels show the central sagittal slice of the respective CT. The position of the axial slice in the left panel is indicated by the dashed blue line in the right panel. The red area in the right panel indicates the positions of all axial slices which contain the SOI and have ground-truth bounding boxes annotated. The heatmaps next to the right panels show the classification score returned by the RetinaNet for each axial slice (light orange = 1.0; black = 0.0). Detections made by the RetinaNet are true positive, if the axial slice has a ground-truth bounding box (red area) and the classification score is > 0.05 (e.g., light orange). **A** Shows an example of a true-positive localization of the SOI; i.e., the highest classification score was returned for a slice which indeed contains the SOI. **B** shows an example of a false-negative localization; i.e., the RetinaNet returned only classification scores < 0.05 even though the SOI is present in one or more slices

For the 379 slices in the test set which included the SOI, the network yielded 338/379 (89.2%) true-positive detections, and 41/379 (10.8%) false-negative detections (Table 2). Examples of a true-positive detection and a false-negative detection are shown in Fig. 4. The median classification score for the 379 test slices that include the SOI was 1.00 [lower quartile (LQ) = 0.98; upper quartile (UQ) = 1.00] (1.0 = perfect score). The median IoU of the predicted bounding boxes and ground-truth bounding boxes in these slices was 0.83 [LQ = 0.76; UQ = 0.88].

**Table 2:** Confusion matrix of RetinaNet detections in the test set. Detections in CT slices which include the SOI and have an IoU > 0.5 with the ground-truth bounding box are true positives. A CT slice which does not include the SOI and for which the RetinaNet did not yield a detection is a true negative

	Detection in slice	No detection in slice
SOI in slice	338 / 379 (89.2%)	41 / 379 (10.8%)
SOI not in slice	51 / 30,467 (0.2%)	30,416 / 30,467 (99.8%)

For the 30,467 (= 30,846—379) slices in the test set that did not include the SOI, the network yielded 51/30,467 (0.2%) false-positive detections and 30,416/30,467 (99.8%) true-negative detections (Table 2). The median classification score for the 51 false-positive detections was 0.88 [LQ = 0.17; UQ = 1.00]. The median classification score for the 30,416 true-negative detections was 0.00 [LQ = 0.00; UQ = 0.00].

### Estimating the location of the SOI

The center of the bounding box from the RetinaNet detection with the highest classification score of all detections in a given CT scan was the estimated location of the SOI for that scan. Estimated locations were compared to ground-truth locations.

In 97/110 (88%) scans of the test set, the estimated location was true positive, i.e., in a slice with a ground-truth location. In 5/110 (5%) scans of the test set, the estimated location was false positive, but in slices directly next to a slice with a ground-truth location. In 8/110 (7%) scans of the test set the location estimate was false negative, because the RetinaNet did not return a positive detection, despite the SOI being present in the scan. The classification score distribution returned by the RetinaNet for a scan of the test set and the slice of the estimated SOI location is included in Fig. 4.

For the 97 true-positive location estimates, the mean (standard deviation (SD)) distance in the axial plane between the estimated location and the true location was 6.0 (3.8) pixels. For the 5 false-positive location estimates, the mean (SD) distance in the axial plane between the estimated location and the closest true location was 7.6 (3.7) pixel. The average number of slices between a false-positive location estimate and the closest true location was 1 slice.

## Discussion

We investigated a deep learning approach based on the state-of-the-art object detection network RetinaNet in order to locate the medial clavicular epiphyseal cartilages through an easy-to-identify proxy structure: the SOI. The dedicated RetinaNet trained in this study achieved an AP of 0.82 for detecting the SOI in all axial CT slices of the test set. Based on the RetinaNet detections, the location of the SOI was estimated correctly in 88% of the CT scans in the test set, the false-positive localizations (5%) being close misses and the false negatives (7%) not being harmful. These results show that the presented localization approach can be used to reliably generate large amounts of annotated data for training and evaluating a dedicated deep neural network for age-assessment, without being limited by expensive and time-consuming manual annotations through medical experts. A large dataset is necessary to train high-performing deep neural networks for any given task [15]. Using the localization approach as a foundation, deep learning–based age estimation has the potential to be more accurate than today’s standard approaches [8, 11, 12]. In addition, the localization approach enables automated end-to-end age assessment without human interaction that only requires a CT scan which includes the medial clavicular epiphyseal cartilages as input.

The presented localization approach enables large annotated datasets for deep learning–based age assessment for multiple reasons. First, in the majority of the test scans (97/110 (88%)), the predicted location of the SOI was in a correct axial slice and only 6.0 pixels away from the ground-truth location on average. Even in the small number of test scans with false-positive detections (5/110 (5%)), the SOI was misplaced by only one slice. The three-dimensional field of view of a deep neural network for age assessment could most likely be chosen large enough, so that the localization in these five respective scans would still be sufficient. Moreover, in all remaining test scans (8/110 (7%)) the RetinaNet did not yield a detection. Although the number of false negatives should be reduced in the future, they are unproblematic for the generation of a dataset for deep learning–based age assessment. False negatives only reduce the number of annotated scans that can be generated from a given amount of unlabeled CT scans. As long as the required number of cases remains feasible, negative detections could also trigger the need for manual annotation of the medial clavicular epiphyseal cartilages through medical experts. This way, every available CT scan including the medial clavicular epiphyseal cartilages may be used as training data for a deep age-assessment network.

To the best of our knowledge, there exists no comparable anatomical landmarking approach for the purpose of locating the medial clavicular epiphyseal cartilages, the sternum, or the clavicles in CT scans. However, anatomic landmarking in medical images is an active research field, and there are a variety of studies which apply deep learning to locate different anatomical structures for distinct purposes [26, 27]. A particular application for anatomic landmarking in medical images, which also shares some conceptual overlap with our study, is the detection of bone fractures. In these studies, deep object detection networks could successfully be trained to detect cracks in bone tissue and to locate fractures in hand and chest radiographs or chest CT scans [27]. Among other areas, one particular network was able to draw a bounding box around the clavicles in radiographic images in case of a present fracture [26].

There are limitations within this study. First, the number of patients (*n* = 202) and CT scans (*n* = 222) was small, because manual ground-truth annotations (*n* = 872 bounding boxes) were time-consuming and data acquisition through the PACS laborious. However, the dataset was deemed large enough to perform this first study. Second, the training set included 100 CT scans from 82 patients; the validation set included 12 CT scans from 10 patients, which means that patient doublets were presented to the RetinaNet in each training epoch. The prevention of doublets is generally considered a quality standard regarding the reference population. The test set used for evaluation did not contain doublets. Third, the dataset is limited to CT images, and no statement about the performance of the automated localization approach for MRI images can be made. CT images were used because CT is the state-of-the-art for forensic questions as it is widely available, quick, cheap, and robust. However, MRI is more desirable for acquiring images in healthy individuals compared to CT, because it spares the individuals from harmful ionizing radiation. But, we believe that the approach can be translated to MRI images in the future. Next, the approach does not differentiate between the left and right sternoclavicular joint and instead locates a proxy structure which includes both joints. As the differentiation of left and right clavicles is crucial in forensic age estimation, expanding the capabilities of the localization in that regard would be an interesting future step. Additionally, the CT scans in this study were originally acquired during the clinical routine for all purposes. Because we were not able to analyze these purposes, there could be a bias in our dataset. Also, the observed false-negative detections may occur systematically and excluding them from a dataset for deep learning–based age assessment could introduce a bias. Furthermore, the thresholds for limiting HU values were derived heuristically and the potential effect of different thresholds on localization performance was not measured. Finally, we did not investigate three-dimensional object detection, even though it would have been natural to the problem of locating the medial clavicular epiphyseal cartilages in a CT scan. However, compared to 3D object detection, 2D object detection is much more common [28] and has a lot of benefits: (a) for the same amount of CT scans, more 2D slices than 3D scans that can be used as training examples, (b) 2D inputs are smaller and allow using smaller networks with fewer parameters, and (c) a wide range of high-performing pretrained models is available for 2D inputs.

## Conclusions

In summary, we demonstrated a robust deep learning–based localization of an anatomical proxy structure to automate the localization of the medial clavicular epiphyseal cartilages. This enables deep learning–based age estimation based on the ossification of the medial clavicular epiphyseal cartilages which might outperform today’s standard methods. The presented localization approach addresses a specific case of a much wider problem concerning machine learning in medicine: human annotations are costly and difficult to acquire, while the lack of annotations poses an enormous bottleneck for machine learning performance [14, 29].

## Figures and Tables

**Fig. 5:**
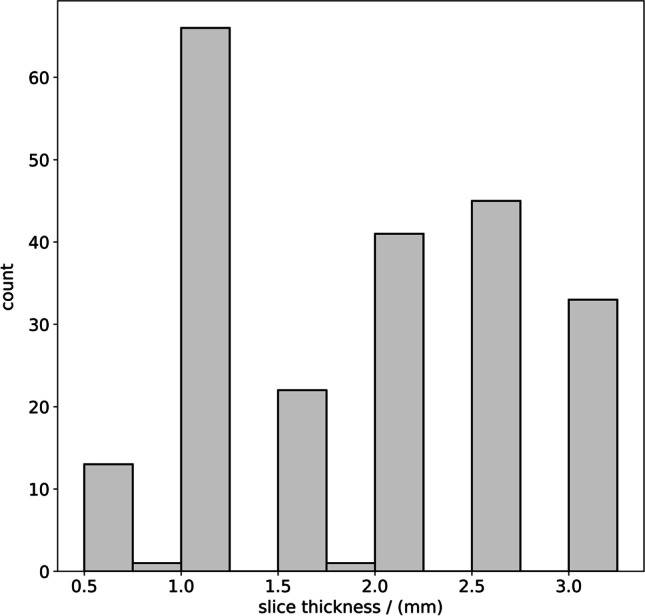
Slice thickness distribution of the 222 chest CT scans

## Data Availability

The datasets generated and analyzed during the current study are not publicly available due to them containing information that could compromise research participant privacy, but are available from the corresponding author on reasonable request and with permission of the institutional review board (Ethics Committee, Medical Faculty, LMU Munich). Custom code described in the manuscript is available in the GitHub repository: https://github.com/pwesp/automated-clavicular-epiphysis-localization.

## Appendix

### CT inclusion and exclusion criteria

The first two inclusion criteria were applied to identify clinical studies in the PACS. Inclusion criteria (PACS):

- Study includes chest CT
- Patient was between 15 and 25 years of age at the time of chest CT acquisition

The remaining 9 inclusion and exclusion criteria are listed below and were applied by inspecting the respective DICOM tags of the chest CT scans. The criteria were applied to each chest CT scan of the identified studies. The list of accepted reconstruction kernels was handcrafted based on all hard kernels we encountered during the retrospective data collection. We did not explicitly filter CT scans based on slice thickness. However, we used slice thickness to include only one chest CT scan per study and thereby keep the number of patients’ duplicates small. Specifically, we selected the chest CT scan with the thinnest slice thickness and discarded all other scans, in case the PACS query yielded multiple eligible chest CT scans for the same study. Nevertheless, patient duplicates exist in the dataset, because some patients were subject to multiple studies. Inclusion criteria (DICOM):

- *Modality* is “CT”
- *ImageOrientationPatient* is [1,0,0,0,1,0]
- *ContrastBolusAgent* is None or “”
- *ConvolutionKernel* is “LUNG,” “BL57f/3,” “BL57d/3,” “I70f/3,” “I70f\3,” [“I70f,” “3”], “I70f/2,” “I70f\2” or [“I70f”, “2”]

Exclusion criteria:

- “patient protocol” in *SeriesDescription.lower()*
- “topogram” in *SeriesDescription.lower()*
- “mpr” in *SeriesDescription.lower()*
- “SPO” in *SeriesDescription*
- “mip” in *SeriesDescription.lower()*

### Slice thickness

The median slice thickness of the 222 CT scans in this study was 2.0 mm, the minimum 0.625 mm, the maximum 3.0 mm, the lower quartile 1.0 mm, and the upper quartile 2.5 mm. The distribution of the 222 slice thickness values is shown in Fig. 5.
